# Ligand Binding Reduces SUMOylation of the Peroxisome Proliferator-activated Receptor γ (PPARγ) Activation Function 1 (AF1) Domain

**DOI:** 10.1371/journal.pone.0066947

**Published:** 2013-06-24

**Authors:** Rolf Diezko, Guntram Suske

**Affiliations:** Institute of Molecular Biology and Tumor Research, Philipps-University Marburg, Marburg, Germany; Institute of Enzymology of the Hungarian Academy of Science, Hungary

## Abstract

Peroxisome proliferator-activated receptor gamma (PPARγ) is a ligand-activated nuclear receptor regulating adipogenesis, glucose homeostasis and inflammatory responses. The activity of PPARγ is controlled by post-translational modifications including SUMOylation and phosphorylation that affects its biological and molecular functions. Several important aspects of PPARγ SUMOylation including SUMO isoform-specificity and the impact of ligand binding on SUMOylation remain unresolved or contradictory. Here, we present a comprehensive study of PPARγ1 SUMOylation. We show that PPARγ1 can be modified by SUMO1 and SUMO2. Mutational analyses revealed that SUMOylation occurs exclusively within the N-terminal activation function 1 (AF1) domain predominantly at lysines 33 and 77. Ligand binding to the C-terminal ligand-binding domain (LBD) of PPARγ1 reduces SUMOylation of lysine 33 but not of lysine 77. SUMOylation of lysine 33 and lysine 77 represses basal and ligand-induced activation by PPARγ1. We further show that lysine 365 within the LBD is not a target for SUMOylation as suggested in a previous report, but it is essential for full LBD activity. Our results suggest that PPARγ ligands negatively affect SUMOylation by interdomain communication between the C-terminal LBD and the N-terminal AF1 domain. The ability of the LBD to regulate the AF1 domain may have important implications for the evaluation and mechanism of action of therapeutic ligands that bind PPARγ.

## Introduction

PPARγ (NR1C3) is a ligand-activated transcription factor that plays an important role in various physiological processes including adipogenesis [1], [2], [3], glucose homeostasis [4] and inflammatory responses [5], [6]. PPARγ binds to enhancers and promoters of target genes as a heterodimer with retinoid X receptor alpha RXRα [7]. Alternative promoter usage yields two PPARγ isoforms (PPARγ1 and PPARγ2) that differ in their N-terminal extension. PPARγ2 contains 30 amino terminal amino acids that are absent in PPARγ1 [8]. Expression of PPARγ2 is largely restricted to adipocytes whereas PPARγ1 is found in several tissues [9] including lower intestine and macrophages.

**Figure 1:**
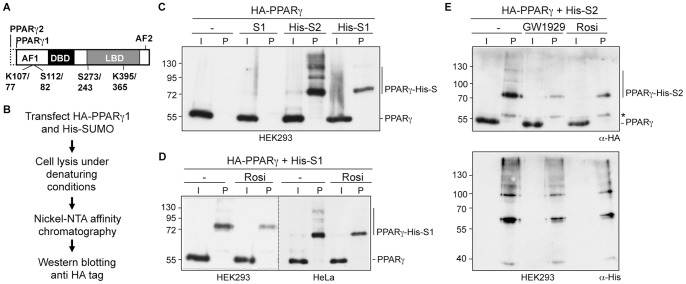
Analyzing SUMOylation of PPARγ. (A) PPARγ domain structure. PPARγ2 differs from PPARγ1 by a 30 amino acid extension at the N-terminus. The activation function 1 and 2 domains (AF1 and AF2), the DNA-binding domain (DBD) and the ligand-binding domain (LBD) are indicated. Positions of lysines (K) and serines (S) refer to PPARγ2 and PPARγ1, respectively. (B) Schematic outline of the experimental procedure for detecting SUMOylated PPARγ1. HA-PPARγ1 was transfected along with untagged SUMO1, His-SUMO1 or His-SUMO2 in HEK293 or HeLa cells. His-SUMO-conjugated proteins were subsequently purified from cell lysates by Ni-NTA affinity chromatography. SUMOylated HA-PPARγ1 was detected by immunoblotting for the HA-tag. (C) SUMOylation of PPARγ was analyzed as outlined in Figure 1B. PPARγ is SUMOylated by His-SUMO1 and His-SUMO2. (D) SUMOylation of PPARγ by His-SUMO1 in HEK293 or HeLa cells was analyzed as outlined in Figure 1B in the absence and presence of 1 µM rosiglitazone. (E) Upper panel: SUMOylation of PPARγ by His-SUMO2 in HEK293 cells was analyzed as outlined in Figure 1B in the absence and presence of 1 µM GW1929 or 1 µM rosiglitazone. The asterisk indicates a cross-reacting protein. Lower panel: To control for loading, the blot was re-probed with an anti His antibody. S1, untagged SUMO1, His-S1 and His-S2, His-tagged SUMO1 and His-tagged SUMO2; I, Input: 1% of cell lysate; P, Ni-pulldown: 90% of cell lysate.

The modular domain structure of PPARγ resembles those of other nuclear receptors and consists of an N-terminal activation function 1 (AF1) domain, a DNA-binding domain (DBD), a C-terminal ligand-binding domain (LBD) and the most C-terminal activation function 2 (AF2) domain (Figure 1A). PPARγ is activated by polyunsaturated fatty acids and certain prostaglandins [10]. Synthetic PPARγ agonists include thiazolidinediones such as rosiglitazone and pioglitazone that ameliorate insulin resistance and lower blood glucose in patients with type 2 diabetes.

PPARγ is subject to several post-translational modifications (reviewed in [11], [12]) including phosphorylation, ubiquitination, O-GlcNAcetylation and SUMOylation that control transcriptional activity and stability. Phosphorylation occurs at serine 112 (S82 in PPARγ1) within the AF1 domain by extracellular signal-regulated kinase 1 or 2 [13] resulting in decreased transcription activity in reporter assays and decreased biological activity. Interestingly, phosphorylation of the amino terminal S112 reduces ligand binding to the C-terminus of PPARγ indicating an interdomain communication between the N-terminal AF1 and the C-terminal LBD/AF2 domains [14]. Another serine in the PPARγ ligand-binding domain (S273) is phosphorylated by cyclin-dependent kinase 5 [15]. Phosphorylation of serine 273 dampens the expression of selected genes such as adiponectin or adipsin and is blocked by rosiglitazone.

Several research groups reported SUMOylation of PPARγ by SUMO1 within the AF1 domain at lysine 107 (lysine 77 in PPARγ1) [16], [17], [18], [19], [20]. Cotransfection of PIAS1 or PIAS2ß enhanced SUMOylation of PPARγ suggesting that PIAS family members are SUMO E3 ligases promoting SUMO attachment to PPARγ [18]. Mutation of the lysine 107/77 increased the transcriptional activity of PPARγ suggesting that SUMOylation induces repression [16], [17], [18]. Yamashita *et al.* [16] reported also an interplay between SUMOylation and phosphorylation of PPARγ2. A serine 112 to alanine mutation reduced SUMOylation, whereas a phospho-mimicking serine 112 to aspartate mutation increased SUMOylation of lysine 107. *In vivo*, PPARγ SUMOylation of lysine 107 is regulated by fibroblast growth factor 21 (FGF21). FGF21 knockout mice show increased PPARγ SUMOylation at lysine 107 concomitant with a decrease of PPARγ target gene expression [21].

Another SUMOylation site has been reported within the LBD of PPARγ1 [22]. According to Pascual *et al.* [22], SUMO1 isoform-specific modification of K365 within the LBD is induced by ligands thereby directing PPARγ to the promoters of inflammatory NF-κB target genes where it inhibits transcription [22]. Specificity of SUMO1 isoform-specific conjugation to K365 by PIAS1 was highlighted as a hallmark of PPARγ SUMOylation [23], [24] thereby demarking it from transrepression mediated by liver x receptors, which are SUMOylated specifically by SUMO2/3 promoted by HDAC4 rather than by PIAS1 [24].

Although SUMOylation of PPARγ is well documented in the current literature, several important aspects including SUMO isoform-specificity and the impact of ligand binding on SUMOylation remain unresolved or contradictory. In this study, we provide a comprehensive analysis of SUMOylation of PPARγ1. We found that PPARγ1 can be SUMOylated by SUMO1 as well as SUMO2 arguing against SUMO1 isoform-specificity. SUMOylation occurred exclusively within the N-terminal AF1 domain predominantly at lysines 33 and 77. Ligand treatment reduced SUMOylation of lysine 33 but not of lysine 77. Mutation of the SUMO attachment sites increased basal as well as ligand-induced transcriptional activation by PPARγ but did not affect PPARγ-mediated transrepression. Lysine 365 within the LBD was not a target for SUMOylation, but was essential for ligand-induced reduction of SUMOylation, activation and transrepression. Collectively, our results suggest that PPARγ ligands negatively regulate SUMOylation by intramolecular communication between the C-terminal LBD and the N-terminal AF1 domain.

## Materials and Methods

### Plasmids

The PPARγ1 expression plasmids pcDNA3-HA-PPARγ1, pcDNA3-HA-PPARγ1 K77R and pcDNA3-HA-PPARγ K365R, and the reporter plasmids *pAox-tk*-luc and *iNOS*-luc were obtained from Christopher Glass. The plasmids pSG5-SUMO1, pSG5-His-SUMO1 [25] and pSG5-His-SUMO2 were a gift from Stefan Müller. The *p(NF-κB)3*-luc reporter plasmid [26] was obtained from Lienhard Schmitz. The HA-PPARγ1 K77/365R double mutant was generated by replacing an *EcoRV-XbaI* fragment of pcDNA3-HA-PPARγ1 K77R with the corresponding fragment from the pcDNA3-HA-PPARγ K365R plasmid. The K33R, K64R, K68R, S82A and S82D mutations were generated by site-directed mutagenesis using the QuikChange mutagenesis kit (Stratagene). Detailed primer information will be provided upon request. Expression plasmids for FLAG- PPARγ1 (1-256) and FLAG- PPARγ1 (247-475) were generated by PCR cloning of appropriate cDNA fragments into a homemade CMV-driven triple-FLAG vector. The Gal4-PPARγ1-LBD expression plasmid was generated by PCR cloning of a mouse PPARγ1 fragment corresponding to amino acids 177 to 475 into the pCMV-BD vector (Stratagene). The K365R mutant fragment was introduced into Gal4-PPARγ1-LBD by restriction cloning. The *5*×*UAS*-luc reporter plasmid pFR-luc was purchased from Stratagene.

### Ni-NTA Pull-down Assays and Western Blotting

HEK293 and HeLa cells were cultured under standard conditions. Cells were seeded at a density of 1×10^6^ cells per 10 cm dish, and after 24 hours transfected with 1.5 µg PPARγ1 and 1.5 µg His-SUMO expression plasmids using FuGene HD (Promega). Twenty-four hours after transfection, cells were treated with 1 µM rosiglitazone (Enzo Life Sciences), 1 µM GW1929 (Tocris Bioscience) or the vehicle as indicated in the figures. Forty-eight hours post transfection, cells were lysed in 1 ml lysis buffer (6 M guanidinium HCl, 0.1 M sodium phosphate buffer pH 8.0, 0.05% Tween 20, 20 mM imidazole), and His-SUMO modified proteins were isolated by incubation with 20 µl of Ni-NTA magnetic agarose beads (Qiagen) for 16 hours at 4°C. Beads were washed three times each with 750 µl buffer A (8 M urea, 0.1 M sodium phosphate buffer pH 8.0, 0.05% Tween 20, 20 mM imidazole) and buffer B (8 M urea, 0.1 M sodium phosphate buffer pH 6.4, 0.05% Tween 20, 20 mM imidazole). After a final washing step with phosphate buffered saline, the beads were boiled in 50 µl SDS sample buffer. Proteins were separated by SDS-PAGE and subsequently transferred on an Immobilon-P membrane (Millipore) for chemiluminescence or on an Immobilon-FL membrane (Millipore) for fluorescence detection according to the manufacturer’s instructions. Primary and secondary antibody incubations were carried out in 1% skim milk for 1 hour each at room temperature. The rat anti-HA antibody (3F10, Roche) was used for chemiluminescence (1∶2000 dilution) and for fluorescence (1∶1000 dilution) detection of HA-PPARγ1 proteins. The anti-FLAG M2 (Sigma), 1∶1000, antibody was used for detection of FLAG-PPARγ (1-256) and FLAG-PPARγ (247-475). Visualization of immunoblots by chemiluminescence was performed with horseradish peroxidase-coupled anti-rat or anti-mouse antibodies (GE Healthcare Life Science, 1∶15,000) followed by incubation with the Immobilon Western chemiluminescent horseradish peroxidase substrate (Millipore). The IRDye 680-labeled anti-rat secondary antibody (LI-COR Biosciences, 1∶2000) was used for quantitative fluorescent detection with the Odyssey Infrared Imager (LI-COR Biosciences).

### Reporter Gene Assays

Cells were seeded on 24-well plates (8×10^4^ cells/well) and cultured for 24 hours prior transfection. For transactivation assays, cells were transfected with 250 ng of reporter plasmid (*Aox-tk*-luc or *5*×*UAS*-luc), 50 ng of expression plasmid (PPARγ mutants or Gal4-PPARγ-LBD) and 0.5 ng of the Renilla luciferase plasmid pRL-CMV (Promega) as internal reference. Twenty-four hours post transfection, 1 µM rosiglitazone or 1 µM GW1929 was added, and cells were incubated for additional 24 hours. For transrepression assays with the *iNOS* promoter, RAW264.7 cells received 500 ng of *iNOS*-luc reporter plasmid, 200 ng of HA-PPARγ expression plasmid and 10 ng of phRL-TK Renilla luciferase plasmid. Forty-two hours post transfection, cells were treated for 6 hours with 1 µg/ml LPS (*E. coli* 0127:B8, Sigma) and/or 1 µM rosiglitazone as indicated in the figures. For transrepression assays with the *3*×*NF-κB* promoter, HeLa cells were transfected with 250 ng of *p(NF-κB)3*-luc reporter plasmid, 50 ng of HA-PPARγ1 expression plasmid and 0.5 ng of the pRL-CMV (Promega) Renilla luciferase plasmid. Twenty-four hours after transfection, cells were treated with 1 µM rosiglitazone for 24 hours. Ten ng/ml interleukin-1ß (Thermo Scientific) was added four hours prior cell lysis. Cells were lysed and firefly and Renilla luciferase activities were determined using the Dual Luciferase Kit (Promega) and the Berthold AutoLumat Plus LB953 multi-tube luminometer.

## Results

### PPARγ is SUMOylated by SUMO1 and SUMO2

SUMO1 isoform-specific modification of PPARγ is portrayed as a hallmark of PPARγ SUMOylation [24], [27]. Close inspection of the studies on PPARγ SUMOylation, however, revealed that SUMOylation of PPARγ by SUMO2 was not addressed. Therefore, we asked whether PPARγ could also be SUMOylated by SUMO2. Since only a very small fraction of the PPARγ protein is SUMOylated at steady-state, we employed a protocol that relies on the enrichment of SUMO conjugates by purification of 6×His-SUMO under denaturing conditions, followed by Western blotting for the protein of interest [28] (Figure 1B). PPARγ was modified by His-SUMO1 as well as by His-SUMO2 (Figure 1C). The absence of any recovered PPARγ upon transfection of untagged SUMO1 confirmed specificity of the PPARγ-SUMO signals. PPARγ was more efficiently modified by SUMO2 than by SUMO1, and several higher molecular weight PPARγ species were visible upon His-SUMO2 transfection indicating multiple SUMO attachment sites or poly SUMO chain formation. We conclude that SUMOylation of PPARγ is not SUMO1 isoform-specific, but that PPARγ is also efficiently modified by SUMO2.

### Ligands Reduce SUMOylation of PPARγ

We investigated SUMOylation of PPARγ in the presence of its synthetic ligand rosiglitazone and the nonthiazolidinedione PPARγ agonist GW1929. Modification of PPARγ by SUMO1 as well as by SUMO2 was reduced in the presence of ligands (Figures 1D and 1E). Reduced SUMOylation of PPARγ upon ligand treatment occurred in HEK293 as well as in HeLa cells. This result is in accordance with the observation of Ohshima *et al.* [18], who found reduced levels of SUMO1-conjugated PPARγ2 in HEK293 cells following rosiglitazone treatment. However, reduced SUMOylation of PPARγ in the presence of rosiglitazone contradicts the result of Pascual *et al.* [22], who reported increased SUMO1 conjugation of transiently expressed PPARγ1 in HeLa cells.

### Ligand Binding to the C-terminal LBD Reduces SUMOylation of Lysine 33 within the N-terminal AF1 Domain

**Figure 2:**
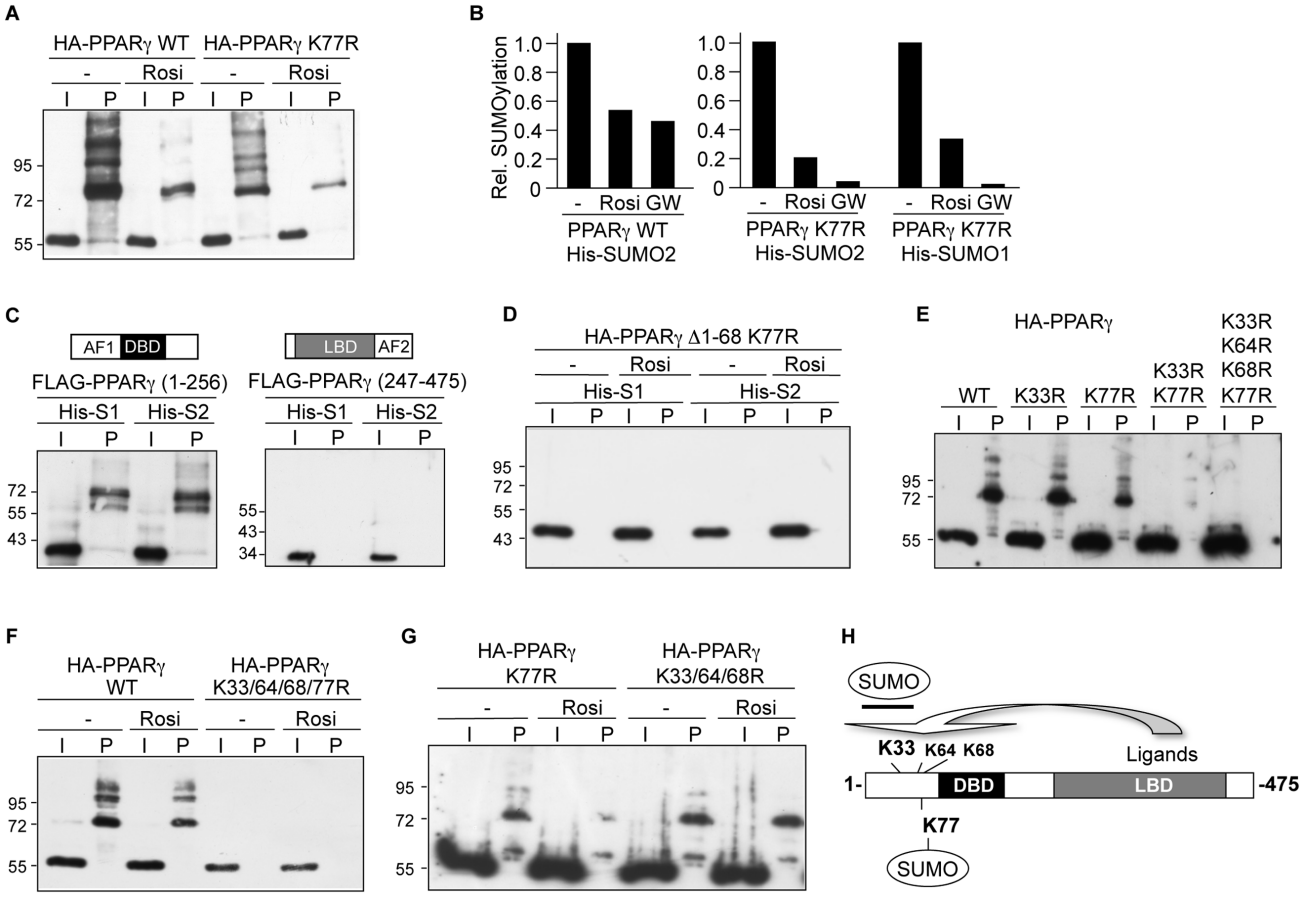
Ligand binding to the C-terminal LBD reduces SUMOylation of lysine 33 within the N-terminal AF1 domain. PPARγ mutants were transfected in HEK293 cells and analyzed for His-SUMO2 or His-SUMO1 modification in the absence and presence of ligands as outlined in the legend to Figure 1. **(**A) SUMOylation of wild type PPARγ and of the PPARγ K77R mutant in the absence and presence of 1 µM rosiglitazone (Rosi). (B) Summary of quantitative Western blot analyses. SUMOylation of wild type PPARγ by His-SUMO2 and of the PPARγ K77R mutant by His-SUMO2 or His-SUMO1 in the absence and presence of rosiglitazone (Rosi) or GW1929 (GW) was analyzed by imager quantification using fluorescence-labeled secondary antibodies. Wild type PPARγ and the PPARγ K77R mutant were analyzed separately. The values obtained for SUMOylated wild type PPARγ or for the PPARγ K77R mutant relative to the input signal in the absence of ligands were arbitrarily set to 1. (C) Analysis of the N-terminal (amino acid 1-256) and the C-terminal domain (amino acid 247-475) of PPARγ for modification by His-SUMO1 or His-SUMO2. (D) Analysis of the PPARγ Δ1-68 K77R mutant for SUMOylation by His-SUMO1 or His-SUMO2 in the absence or presence of rosiglitazone. (E) Analysis of the PPARγ mutants PPARγ K33R, PPARγ K77R, PPARγ K33/77R and PPARγ K33/64/68/77R for modification by His-SUMO2. (F) Analysis of the PPARγ K33/64/68/77R mutant for modification by His-SUMO2 in the absence or presence of rosiglitazone. (G) Analysis of the PPARγ K77R and PPARγ K33/64/68R mutants for modification by His-SUMO2 in the absence or presence of rosiglitazone. **(**H) Model depicting interdomain communication regulating SUMOylation of PPARγ. Ligands reduce SUMOylation of K33 and potentially of K64 and K68 but not of K77.

PPARγ1 contains a perfect SUMOylation consensus sequence (ψKXE, ψ represents a hydrophobic amino acid) at K77 within the N-terminal AF1 domain. We analyzed the PPARγ mutant in which K77 is replaced by an arginine residue (Figure 2A). Compared to wild type PPARγ, the amount of SUMO2-modified PPARγ K77R protein was much less in the absence of ligand (Figure 2A) supporting the previous assignment of K77 as a SUMO attachment site [16], [18]. However, the PPARγ K77R protein was still SUMOylated indicating the existence of an additional SUMO site. Moreover, treatment with rosiglitazone further strongly decreased SUMOylation of the PPARγ K77R mutant (Figure 2A) suggesting that SUMOylation of a lysine other than K77 was negatively affected by ligand treatment. This conclusion was further supported by independent quantitative Western blot analyses using fluorescence-labeled secondary antibodies followed by imager quantification (Figure 2B). Rosiglitazone as well as GW1929 reduced SUMO2-modification of the PPARγ K77R mutant. Moreover, also modification of the PPARγ K77R mutant by the SUMO1 isoform was reduced in the presence of ligands (Figure 2B). In conclusion, there is also no SUMO-isoform specificity with respect to ligand-induced reduction of PPARγ SUMOylation.

To map the additional SUMO site(s) in PPARγ, we analyzed at first the N- and C-terminal domains of PPARγ on their own. The N-terminal domain of PPARγ comprising the AF1 and the DNA-binding domains (amino acids 1–256) but not the C-terminal domain comprising the LBD and the AF2 domain (amino acids 247–475) was SUMOylated (Figure 2C). Next, we analyzed a series of N-terminal PPARγ1 deletion mutants. These experiments revealed that PPARγ1 was SUMOylated exclusively within the N-terminal AF1 domain, exemplified by the PPARγ Δ1-68 K77R mutant that was neither modified by SUMO1 nor by SUMO2 in the absence or in the presence of rosiglitazone (Figure 2D).

The amino acid sequence 1–68 of PPARγ contains three lysines at positions 33 (DIK_33_P), 64 (DYK_64_Y) and 68 (DLK_68_L) that fit the recently uncovered inverted SUMOylation consensus motif D/EXKY/P [29]. We analyzed PPARγ mutants in which these lysines were replaced by arginines. By this analysis we identified lysine 33 as an additional SUMO attachment site (Figure 2E). Most significantly, the PPARγ K33/77R double mutant showed only a very weak residual SUMOylation signal that, however, was completely abolished when K64 and K68 are mutated additionally (Figure 2E and F). Finally, we identified the SUMO sites in PPARγ that were affected by ligands. Rosiglitazone treatment reduced SUMOylation of wild type PPARγ and of the PPARγ K77R mutant (Figures 2A, B and G). This result implied that ligands affected SUMOylation of K33 and potentially also of K64 and K68. To explore whether rosiglitazone also reduced SUMOylation at K77, we compared SUMOylation of the PPARγ K33/64/68R triple mutant in the absence and presence of ligand. Rosiglitazone did not affect SUMOylation of the PPARγ K33/64/68R triple mutant (Figure 2G) showing that SUMOylation of K77 is not affected upon ligand treatment. In conclusion, our results strongly suggest that ligand binding to the C-terminal LBD of PPARγ reduces SUMOylation of the N-terminal AF1 domain at K33 (Figure 2H).

### PPARγ Serine 82 Mutations do not Affect SUMOylation

**Figure 3:**
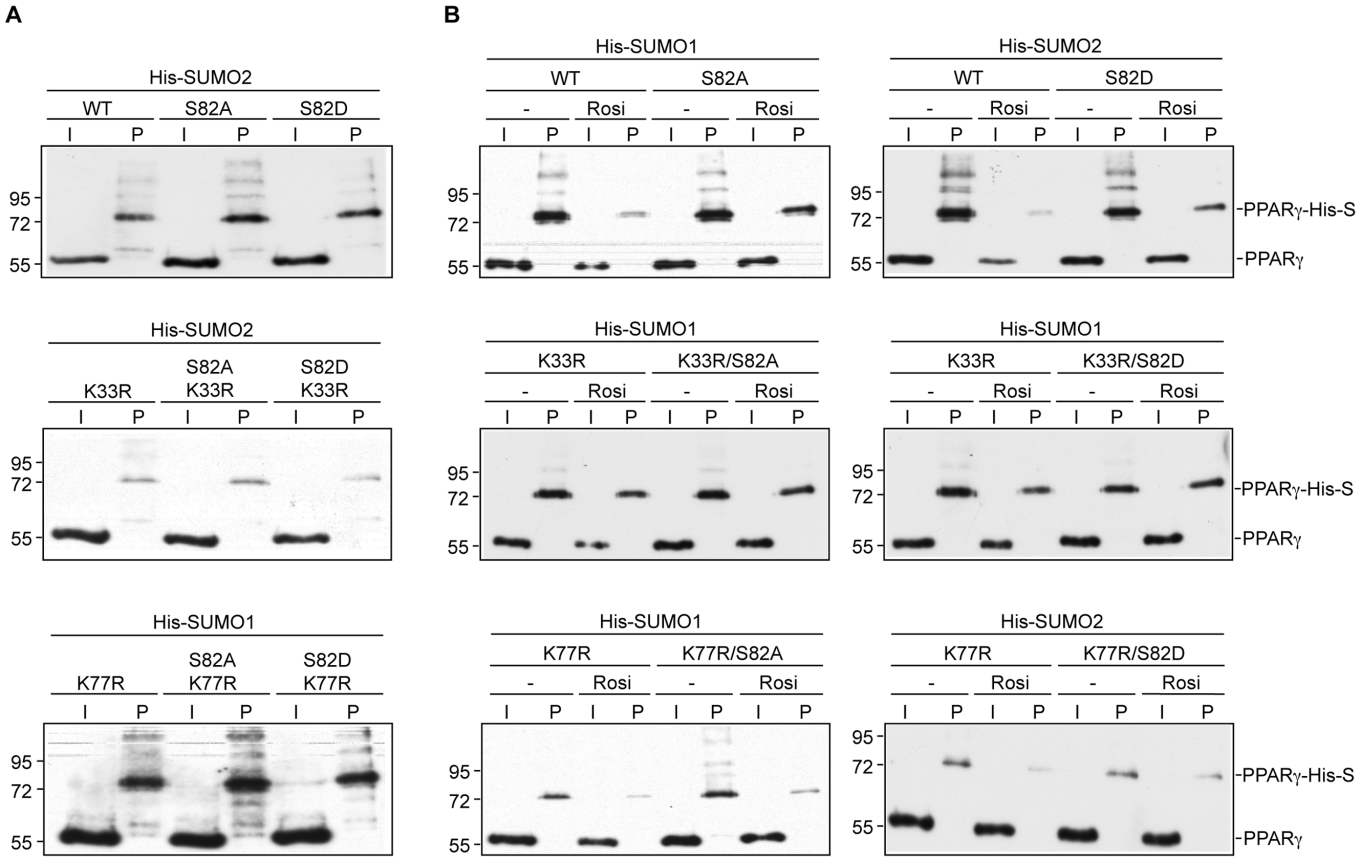
PPARγ S82A and S82D mutations do not affect SUMOylation. The indicated PPARγ K33R, K77R, S82A, S82D, K33R/S82A, K33R/S82D, K77R/S82A and K77R/S82D mutants were transfected in HEK293 cells and analyzed for SUMO modification in the absence and presence of ligands as outlined in the legend to Figure 1. (A) The phosphorylation blocking S82A and the phosphorylation mimicking S82D mutations did not significantly affect SUMOylation of PPARγ at K33 and K77. (B) PPARγ S82A and S82D mutations did not affect rosiglitazone-induced reduction of PPARγ SUMOylation at K33.

Lysine 77 is located within a phosphorylation-dependent SUMOylation motif [30], [31] (IK_77_VEPAS_82_P). Serine 82 (S112 in PPARγ2) is phosphorylated by MAP kinases and a previous report provided evidence that phosphorylation of S112 increases SUMOylation at K107 in PPARγ2 [16]. We asked whether S82 phosphorylation blocking or mimicking mutations (PPARγ1 S82A and PPARγ1 S82D) affect SUMOylation of PPARγ1 in the absence or presence of ligands. We introduced both types of serine 82 mutations into wild type PPARγ and into the PPARγ K33R and PPARγ K77R mutants, and analyzed the various PPARγ lysine/serine mutants for SUMOylation by SUMO1 and SUMO2 (Figure 3). Neither the S82A nor the S82D mutation significantly affected SUMOylation of wild type PPARγ, PPARγ K33 or PPARγ K77 in the absence of ligands (Figure 3A). Rosiglitazone treatment reduced SUMOylation of wild type PPARγ and of all PPARγ mutants in which K33 was unchanged, irrespectively whether serine 82 was mutated to alanine or aspartate (Figure 3B, top and bottom panels). In contrast, rosiglitazone did not affect SUMOylation of the PPARγ K33R/S82A and of the PPARγ K33R/S82D double mutants (Figure 3B, middle panels). In summary, these results do not support the possibility that phosphorylation of serine 82 regulates SUMOylation of lysine 77. Importantly, however, the analysis of the various PPARγ lysine/serine double mutants further corroborates the conclusion that rosiglitazone specifically regulates SUMOylation of K33 but does not affect SUMOylation of K77.

### Lysine 365 is not SUMOylated but is Essential for Ligand-induced Reduction of SUMOylation

**Figure 4:**
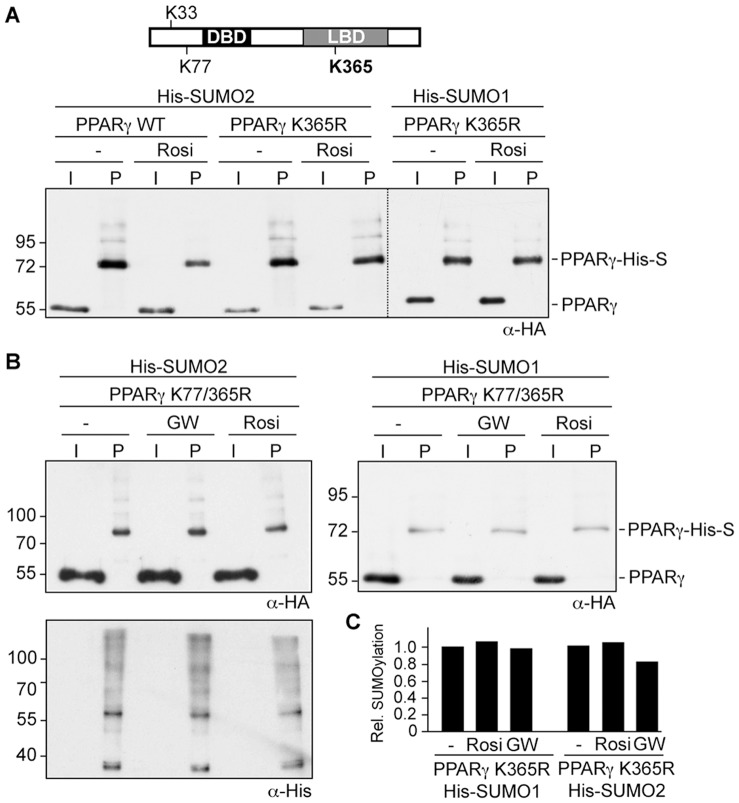
Lysine 365 within the LBD is essential for ligand-induced reduction of PPARγ SUMOylation. **(**A) (B) and (C) The PPARγ K365R (A) and PPARγ K77/365R (B) mutants were transfected in HEK293 cells and analyzed for His-SUMO2 and His-SUMO1 modification in the absence and presence of ligands as outlined in the legend to Figure 1. The blot shown in the upper left panel of figure 4B was re-probed with an anti-His antibody to control for loading. (C) Summary of quantitative Western blot analysis. SUMOylation of the PPARγ K365R mutant in the absence or presence of rosiglitazone or GW1929 was analyzed by an independent quantitative Western blot analysis using fluorescence-labeled secondary antibodies. The values obtained for SUMOylated PPARγ K365R relative to the input signal in the absence of ligands were arbitrarily set to 1.

Lysine 365 located within the C-terminal LBD of PPARγ1 is also embedded in a SUMO consensus motif (PK_365_FE), and it was previously reported that SUMO1 modification of K365 is induced by rosiglitazone [22]. Our results do not support the assignment of K365 as a SUMO attachment site as rosiglitazone treatment of the PPARγ K33/64/68/77 quadruple mutant did not result in any SUMOylation signal (Figure 2F). However, since ligand binding reduced SUMOylation of the AF1 domain, we asked whether mutation of K365 would affect SUMOylation of the AF1 domain. We analyzed PPARγ mutants in which K365 was replaced by arginine (Figure 4). SUMOylation of the PPARγ K365R mutant in the absence of ligands was similar to wild type PPARγ (Figure 4A). Strikingly, however, treatment with rosiglitazone did not reduce SUMO modification of the PPARγ K365R mutant by SUMO2 or SUMO1 (Figure 4A). This result was corroborated by the analysis of the PPARγ K77/365R double mutant. PPARγ K77/365R was still SUMOylated but SUMOylation did not change upon ligand treatment (Figure 4B). These results were further substantiated by an independent quantitative Western blot analysis using fluorescence-labeled secondary antibodies. Neither SUMO1 nor SUMO2 modification of the PPARy365R mutant was reduced upon rosiglitazone or GW1929 treatment (Figure 4C). Collectively, these findings suggest that K365 is not SUMOylated either in the absence or presence of ligands. However, the K365 residue appears to be essential for ligand-induced reduction of SUMOylation of the AF1 domain.

### SUMOylation of PPARγ Represses its Activation Function but does not Affect its Transrepression Function

**Figure 5:**
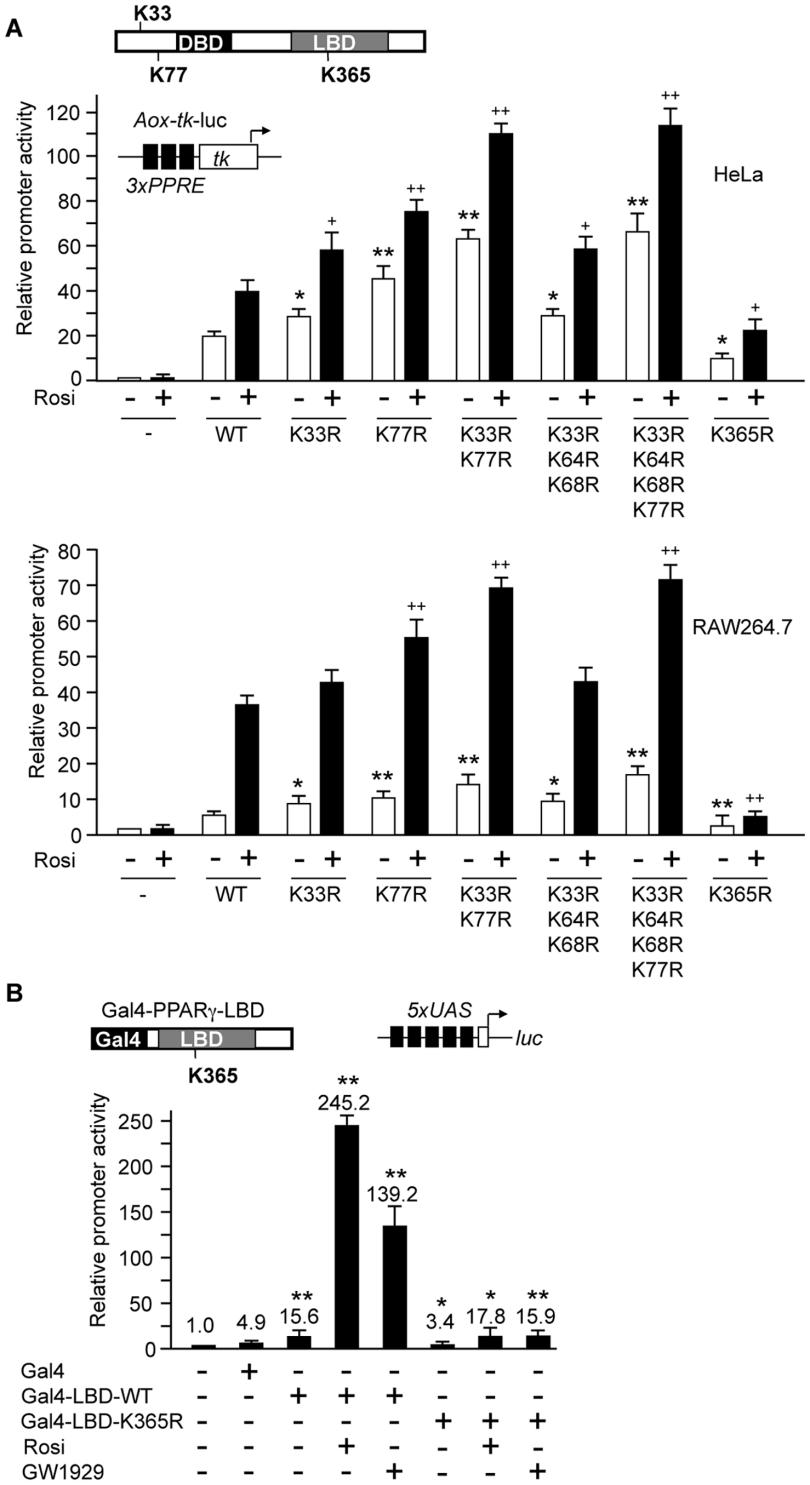
Transcriptional activity of PPARγ mutants. (A) HeLa (top) and RAW264.7 (bottom) cells were transfected with the *Aox-tk* luciferase reporter construct along with the indicated PPARγ1 lysine mutants. Twenty-four hours after transfection, cells were treated with 1 µM rosiglitazone (+) or the vehicle (-), and incubated for additional 24 hours. The reporter activity in the absence of PPARγ was arbitrarily set to 1. Error bars are mean +/− SD. Statistical significance of activation by PPARγ mutants compared to wild type PPARγ in the absence (*) or presence (^+^) of rosiglitazone was calculated using the Student´s t-test. * and ^+^, p<0.05; ** and ^++^, p<0.005. (B) HEK293 cells were transfected with a *5*×*UAS*-driven luciferase reporter along with expression constructs for Gal4 or Gal4-PPARγ-LBD fusions as indicated. Twenty-four hours after transfection, cells were treated with 1 µM rosiglitazone (Rosi) or 1 µM GW1929 for additional 24 hours. The reporter activity in the absence of Gal4 fusions was arbitrarily set to 1. Error bars are mean +/− SD. Statistical significance of activation by Gal4-LBD and Gal4-LBD-K365R compared to Gal4 was calculated by the Student´s t-test. *, p<0.05; **, p<0.005.

To analyze the impact of the individual SUMOylation sites on the transactivation capacity of PPARγ we performed reporter gene assays in HeLa and RAW264.7 cells using a luciferase reporter gene driven by three copies of the acyl CoA oxidase PPARγ response element linked to the *tk* promoter (*Aox-tk-*luc) [5]. In HeLa cells, wild type PPARγ activated the *Aox-tk* construct approximately 20-fold, which increased up to 40-fold upon rosiglitazone treatment (Figure 5, top). Compared to wild type PPARγ, activation by the PPARγ K33R and the PPARγ K77R was approximately 1.5-fold and 2-fold higher in the absence as well as in the presence of rosiglitazone. Additional 2-fold activation was obtained with the PPARγ K33/77R double mutant (Figure 5A, top). In RAW264.7 cells, the fold-induction rate by rosiglitazone in the presence of wild type PPARγ was significantly higher than in HeLa cells (Figure 5A, bottom). Still the PPARγ K33R, PPARγ K77R and PPARγ K33/77R mutants showed increased activation in the absence and presence of rosiglitazone (Figure 5A, bottom). Similar results were obtained when we treated the cells with the GW1929 ligand (data not shown). In conclusion, SUMOylation of both lysine residues, K33 and K77, represses PPARγ1-dependent activation. We also analyzed the PPARγ K33/64/68R triple mutant and the PPARγ K33/64/68/77R quadruple mutant. Activation by the PPARγ K33/64/68R triple mutant was similar to the PPARγ K33R mutant and activation by the PPARγ K33/64/68/77R quadruple mutant was similar to the PPARγ K33/77R double mutant (Figure 5A). This result suggests that SUMOylation of K64 and K68, which was negligible as compared to SUMOylation of K33 and K77 (see Figure 2) does not markedly influence the activation capacity of PPARγ.

We also analyzed the PPARγ K365R mutant for activation of the *Aox-tk* promoter. The PPARγ K365R mutant was much less active in the absence as well in the presence of ligand. Strongly reduced activity of the PPARγ K365R mutant contradicts the results of Pascual *et al.* [22] who reported similar activation of the *Aox-tk* luciferase construct by wild type PPARγ and by the PPARγ K365R mutant. To finally clarify whether the K365R mutation affects the activation function of PPARγ, we analyzed the K365R mutation also in another experimental setting. We fused the PPARγ wild type LBD and the PPARγ K365R mutant LBD to the DNA binding domain of the yeast transcription factor Gal4, and analyzed the activity of the Gal4-PPARγ-LBD fusion proteins on a Gal4-responsive promoter (Figure 5B). The Gal4-PPARγ-LBD-wt protein activated transcription from the *5*×*Gal4*-promoter up to 50-fold and 28-fold in the presence of rosiglitazone or GW1929, respectively. Activation by the Gal4-PPARγ-LBD-K365R mutant, however, was much lower in the absence and presence of ligands (Figure 5B). This result supports the conclusion that the K365R mutation within the PPARγ-LBD impairs LBD activity.

**Figure 6:**
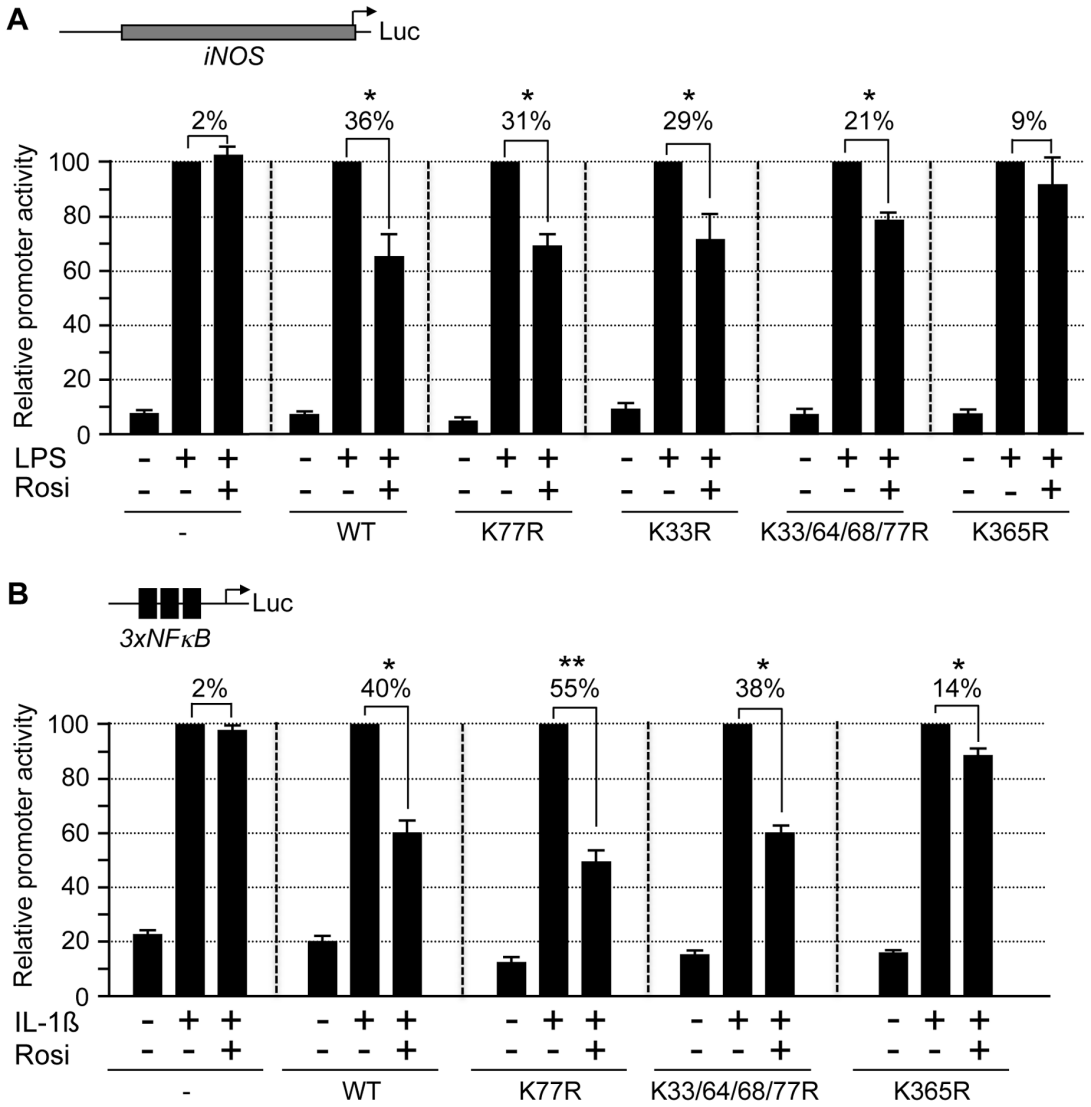
Transrepression activity of PPARγ mutants. (A) RAW264.7 macrophages were transfected with the *iNOS* luciferase reporter plasmid along with PPARγ mutants. Forty-two hours after transfection, cells were treated for 6 hours with 1 µg/ml LPS and 1 µM rosiglitazone (Rosi) as indicated. The reporter activities in the presence of LPS were set to 100% promoter activity. (B) Hela cells were transfected with the *3xNF-κB* luciferase reporter plasmid along with PPARγ mutants. Twenty-four hours after transfection, cells were treated with 1 µM rosiglitazone (Rosi). Four hours prior lysis, 10 ng/ml interleukin-1β (IL-1ß) was added as indicated. The reporter activities obtained by interleukin-1ß stimulation were set to 100% promoter activity. Error bars are mean +/− SD. Statistics was performed using Student´s t-test. *, p<0.05; **, p<0.005.

PPARγ ligands can modulate inflammatory signaling by repressing the induction of inflammatory genes without directly binding to their promoters [5]. This transrepression activity of PPARγ ligands can be monitored by their ability to repress lipopolysaccharide (LPS)-induced activation of the mouse *inducible nitric oxide synthase* (*iNOS*) promoter in RAW264.7 macrophages [5], [22]. We tested PPARγ mutants for their ligand-dependent transrepression activity by cotransfecting an *iNOS* promoter-driven luciferase construct along with PPARγ expression constructs in RAW264.7 cells (Figure 6A). LPS treatment activated the *iNOS* promoter up to 13-fold. Rosiglitazone did not affect activation in absence of PPARγ but inhibited activation by approximately 36% in the presence of PPARγ. All PPARγ variants with K33R or K77R mutations including the SUMOylation-deficient PPARγ K33/64/68/77R quadruple mutant exerted also transrepression activities although to a slightly lesser extent (Figure 6A). This result suggests that SUMOylation of PPARγ is not absolutely essential for repressing LPS-induced activation of the *iNOS* promoter. We also tested the PPARγ K365R mutant in this transrepression assay. The PPARγ K365R mutant failed to significantly mediate repression of the *iNOS* promoter further supporting the conclusion that the K365R mutation impairs PPARγ-LBD activity.

Previously, it was shown that rosiglitazone inhibits promoter activation by NF-κB in the presence of PPARγ [32]. Therefore, we also analyzed the SUMOylation-deficient PPARγ K33/64/68/77R and the PPARγ K365R mutants for their ability to mediate repression of an NF-κB-specific reporter construct that was activated by interleukin-1ß treatment (Figure 6B). Wild type PPARγ as well as the SUMOylation-deficient PPARγ K33/64/68/77R mutant inhibited NF-κB activation by approximately 40% upon rosiglitazone treatment. The PPARγ K365R mutant, however, retained only residual repression activity (Figure 6B).

Collectively, these results strongly suggest that SUMOylation negatively affects activation functions but is largely dispensable for transrepression activity of PPARγ. Lysine 365 is not SUMOylated. However, it is essential for PPARγ ´s activation and transrepression functions.

## Discussion

Previous studies on SUMOylation of PPARγ were rather incomplete or yielded contradictory results. In this study, we showed that PPARγ can be modified by SUMO1 and by SUMO2 within the N-terminal AF1 domain, and we finally mapped the major SUMOylation sites to lysine 33 and 77. SUMOylation within the N-terminal AF1 domain was negatively regulated by ligand binding to the C-terminal LBD affecting activation but not transrepression functions of PPARγ. Interestingly, ligand binding to PPARγ reduced specifically SUMOylation of lysine 33 embedded in the inverted SUMO consensus site [D/E]xKY/P [29], but not of lysine 77 embedded in the classical SUMO consensus site ψKXE. Whether reduced SUMOylation of lysine 33 reflects impaired SUMOylation or, alternatively, enhanced de-SUMOylation remains unclear.

Reduced SUMOylation of PPARγ after ligand treatment is in line with the report of Ohshima *et al.* [18] who found that the amount of SUMO1-conjugated PPARγ2 is lower in HEK293 lysates of rosiglitazone-treated cells. Yet, Pascual *et al.* [22] reported increased SUMOylation of transfected PPARγ1 in HeLa cells following rosiglitazone treatment. Importantly, both studies had not entirely mapped the PPARγ SUMO attachment sites and therefore could not include appropriate controls in their analysis. We believe that our mutational analysis finally clarified the effect of ligands on SUMOylation. Ligand binding to the C-terminal PPARγ LBD reduces SUMOylation of the N-terminal AF1 domain. A future goal should be to define the SUMOylation pattern of PPARγ in primary cells under relevant physiologic and pathologic conditions.

Interestingly, it was reported that, *vice versa*, the AF1 domain can also affect the LBD domain as phosphorylation of the PPARγ AF1 domain at serine 112 by MAP kinase reduced ligand binding affinity to the C-terminal part [14]. Thus, our results lend further credence to the concept of an intramolecular communication between the C- and N-terminal PPARγ domains. How the interplay between the N-terminal AF1 domain and the C-terminal LBD is achieved mechanistically is unknown. The structure of the AF1 domain encompassing the SUMOylation sites was not resolved in the crystallized intact PPARγ-RXRα nuclear receptor complex on DNA [33], and no direct interaction between the AF1 domain and the LBD of PPARγ was detected [14]. Potentially, ligand binding induces allosteric changes that may affect the accessibility of K33 for SUMO-modifying enzymes. An alternative intriguing idea would be that SUMO modification of the AF1 domain mediates a direct interaction between the N-terminal and the C-terminal PPARγ domains. In line with this idea, inspection of the PPARγ LBD revealed several SUMO-interaction motifs. Unfortunately, inefficient *in vitro* SUMOylation of PPARγ impeded interaction studies of SUMOylated N-terminal PPARγ fragments with the C-terminal LBD domain.

Lysine 77/107 is in close proximity to serine 82/112 constituting a phosphorylation-dependent SUMOylation motif (consensus: ψKXEXXSP) [30], [31]. However, neither a serine 82 blocking (S82A) nor a mimicking mutant (S82D) affected SUMOylation of lysine 77 or lysine 33 suggesting that phosphorylation and SUMOylation of the PPARγ1 isoform are uncoupled. Notably, Yamashita *et al.* [16] reported phosphorylation-dependent SUMOylation of the PPARγ2 isoform. Whether phosphorylation-dependent SUMOylation of lysine 77/107 is PPARγ2 isoform-specific -we only analyzed PPARγ1 mutants- or whether differences in the experimental setting account for the different results remain unsolved.

Ligand-activated PPARγ is recruited to promoters of inflammatory genes where it inhibits transcription by preventing proteasome-mediated clearance of repressive nuclear receptor corepressor (N-CoR) complexes. It was reported that the initial step of this pathway involves ligand-induced SUMO1 conjugation to K365 within the PPARγ ligand-binding domain [22]. Accordingly, ligand-induced SUMOylation of PPARγ at lysine 365 specifically by the SUMO1 isoform is repeatedly portrayed in many reviews [1], [11], [12], [23], [34], [35], [36], [37], [38], [39]. Our results do not support the assignment of K365 as a SUMOylation target site as we did not detect any residual SUMOylation of the PPARγ K33/64/68/77R mutant protein neither in the absence nor in the presence of ligands. However, K365 affected SUMOylation of PPARγ indirectly as it prevented ligand-induced reduction of SUMOylation at K33. The PPARγ K365R mutant was much less responsive to rosiglitazone indicating that the K365R mutation affects LBD activity. In line with this finding, it was reported that mutation of K395 in PPARγ2 (corresponding to K365 in the PPARγ1 isoform) also impaired rosiglitazone-induced positive transcriptional activity of PPARγ [19]. Impaired LBD activity of the PPARγ K365R mutant readily explains why (i) rosiglitazone treatment did not affect SUMOylation, (ii) did only weakly activate PPARγ-dependent transcription and (iii) did barely mediate rosiglitazone-induced transrepression. Our results imply that SUMOylation of K365 is not involved in transrepression by PPARγ, but do not necessarily challenge the conclusion of Pascual *et al.* [22] that the SUMOylation machinery is generally required for PPARγ-dependent transrepression. However, how SUMOylation acts in this pathway remains to be uncovered. Notably, several proteins involved in this pathway such as N-CoR [40] and the transducin beta-like proteins TBL1-TBLR1 [41] are also targets of SUMOylation.

Taken together, in this study we unambiguously assigned the SUMOylation sites of PPARγ to lysine residues within the AF1 domain and provide evidence that ligand binding to the C-terminal LBD affects the function of the N-terminal AF1 domain by altering SUMOylation. Thus, our results may have important implications for the evaluation and mechanism of action of therapeutic agonists and antagonists that bind PPARγ.
